# Effects of lactotripeptide ingestion and physical activity intervention on the fatigue status of middle-aged and older adults: a randomized controlled trial

**DOI:** 10.1038/s41598-023-41669-2

**Published:** 2023-09-21

**Authors:** Masaki Yoshioka, Tomoko Kaneko, Karen Yoneko, Masahiro Matsui, Shoya Mori, Natsumi Nishitani, Qin Wenxing, Kei Ouchi, Risa Yasuda, Hayate Namatame, Tomohito Sato, Jiyeon Park, Yoshio Nakata, Seiji Maeda, Keisei Kosaki

**Affiliations:** 1 Graduate School of Comprehensive Human Sciences, University of Tsukuba, 1-1-1 Tennodai, Tsukuba, Ibaraki 305-8574 Japan; 2 Japan Society for the Promotion of Science, 5-3-1 Kouzimachi, Chiyoda-ku, Tokyo, 102-8472 Japan; 3 Institute of Health and Sport Sciences, University of Tsukuba, 1-1-1 Tennodai, Tsukuba, Ibaraki 305-8574 Japan; 4 Faculty of Sport Sciences, Waseda University, 2-579-15 Mikajima, Tokorozawa, Saitama 359-1192 Japan

**Keywords:** Health care, Medical research

## Abstract

This randomized controlled trial aimed to investigate the effects of eight weeks of lactotripeptide (LTP) ingestion, physical activity (PA) intervention, and combined intervention on the fatigue status of middle-aged and older adults. A total of 78 middle-aged and older adults (63 ± 8 years of age) were randomly assigned to four groups: placebo, LTP, placebo with PA intervention (placebo + PA), and LTP with PA intervention (LTP + PA). All participants ingested the placebo or LTP tablets daily (three tablets/day). The placebo + PA and LTP + PA groups participated in a weekly supervised exercise class and were instructed to increase their moderate- to vigorous-intensity PA at home. The visual analog scale, Brief Fatigue Inventory, Profile of Mood States second edition (POMS2), and Beck Depression Inventory second edition (BDI-II) were administered before and after the intervention. No significant interactions or main effects were observed between LTP ingestion and PA intervention on any of the fatigue scales. The main-effect analyses revealed that the PA intervention improved the total mood disturbance score of the POMS2 (*F* = 5.22, *P* = 0.03) and BDI-II score (*F* = 4.81, *P* = 0.03). After the post hoc paired comparisons, the total mood disturbance and BDI-II scores improved more with the combined intervention than with the PA intervention alone (percentage difference between the effect of combined intervention and PA intervention alone was 3.7% for total mood disturbance score and 13.7% for BDI-II score). The present study suggests that eight weeks of LTP ingestion and PA intervention did not have a significant effect on fatigue status. However, the PA intervention improved mood status and depressive symptoms, and these effects were enhanced by LTP ingestion.

## Introduction

Fatigue is a complex medical and public health problem^1–3^. It is estimated that more than two million Americans have chronic fatigue syndrome, many of whom remain undiagnosed^4^. According to a survey by the Ministry of Health, Labor and Welfare, 60% of the Japanese population felt fatigued, at least to some extent^5^. Population-based surveys also suggest that middle-aged and older adults are at a higher risk of chronic fatigue syndrome than other age groups^1,2^. Fatigue can hinder activities of daily living and quality of life in older adults^6^. Therefore, effective health interventions are urgently needed to prevent and improve chronic fatigue in middle-aged and older adults.

Valine–proline–proline (VPP) and isoleucine–proline–proline (IPP), collectively referred to as lactotripeptides (LTP), have been isolated from casein milk protein through the proteolytic action of lactic acid and bacteria. LTP has an inhibitory effect on angiotensin-converting enzymes and is safe in cases of overdose^7,8^. We previously reported that eight weeks of LTP ingestion improved arterial stiffness, endothelial function, and cerebral blood flow velocity in middle-aged and older adults^9–11^. In addition, LTP ingestion may be effective in mitigating fatigue, as an observational study reported that endothelial function is associated with fatigue status^12^. One study reported that a single dose of LTP temporarily alleviated fatigue in middle-aged and older men^13^. However, the effects of long-term LTP ingestion on fatigue have not yet been determined.

World Health Organization guidelines on physical activity (PA) and sedentary behavior (SB) indicate that PA provides significant health benefits^14^. Previous studies have reported that more time spent in PA, especially moderate- to vigorous-intensity PA (MVPA), is beneficially associated with fatigue status in various populations^15–18^. These findings indicate that PA intervention may be effective in improving fatigue status. Additionally, interventions that combine increasing PA and nutritional supplementation may have additional effects on quality of life and depressive symptoms^19,20^. Based on these reports, PA intervention combined with LTP ingestion may also improve fatigue status more than that observed with either treatment alone.

Therefore, this study aimed to investigate the effects of LTP ingestion, PA intervention, and combined intervention on the fatigue status of middle-aged and older adults. We hypothesized that LTP ingestion and PA intervention would provide greater benefits in improving fatigue status than placebo ingestion, LTP ingestion alone, or PA intervention alone.

## Results

Table 1 presents the characteristics of the participants in the four intervention groups. No significant group differences were found in terms of age, sex, height, education, living arrangement, work, family income, marital status, postmenopausal status, or medication use. Supplement compliance showed significant group differences; however, the values were high in all groups (> 95%). No side effects of LTP ingestion were reported by participants throughout the study period. The participation rate in a weekly supervised session was 92 ± 3% in the placebo + PA group and 93 ± 5% in the LTP + PA group.

**Table 1:** Participant characteristics (n = 78).

	Placebo (n = 20)	LTP (n = 20)	Placebo + PA (n = 19)	LTP + PA (n = 19)	*P*
Age, years	63 ± 2	62 ± 2	64 ± 2	63 ± 2	0.94
Men, n (%)	6 (30)	7 (35)	6 (32)	6 (32)	0.99
Height, cm	158 ± 2	158 ± 1	158 ± 1	161 ± 2	0.62
Education, n (%)					0.46
< 9 years	0 (0)	0 (0)	1 (5)	0 (0)	
< 13 years	6 (30)	2 (10)	6 (32)	4 (21)	
< 15 years	5 (25)	8 (40)	7 (37)	8 (42)	
≥ 15 years	7 (35)	10 (50)	5 (26)	6 (32)	
Missing	2 (10)	0 (0)	0 (0)	1 (5)	
Living arrangement, n (%)					0.20
With others	18 (90)	19 (95)	18 (95)	15 (79)	
Alone	2 (10)	0 (0)	1 (5)	1 (5)	
Missing	0 (0)	1 (5)	0 (0)	3 (16)	
Work, n (%)					0.79
Not working	5 (25)	3 (15)	6 (32)	3 (16)	
Homemaker	3 (15)	7 (35)	3 (16)	6 (32)	
Full-time job	4 (20)	5 (25)	5 (26)	4 (21)	
Part-time job	5 (25)	5 (25)	3 (16)	2 (11)	
Others	1 (5)	0 (0)	1 (5)	2 (11)	
Missing	2 (10)	0 (0)	1 (5)	2 (11)	
Family income (JPY), n (%)					0.92
< 3 million	5 (25)	1 (5)	4 (21)	6 (32)	
3 million ≤, < 5 million	5 (25)	5 (25)	3 (16)	3 (16)	
5 million ≤, < 7 million	3 (15)	6 (30)	4 (21)	5 (26)	
7 million ≤, < 10 million	2 (10)	4 (20)	3 (16)	1 (5)	
10 million ≤	2 (10)	1 (5)	2 (11)	2 (11)	
Missing	3 (15)	3 (15)	3 (16)	2 (11)	
Marriage, n (%)					0.90
Yes	17 (85)	17 (85)	17 (90)	18 (95)	
No	2 (10)	2 (10)	1 (5)	0 (0)	
Missing	1 (5)	1 (5)	1 (5)	1 (5)	
Post-menopause, n (%)					0.48
Yes	12 (86)	12 (92)	12 (92)	12 (92)	
No	2 (14)	0 (0)	1 (8)	0 (0)	
Missing	0 (0)	1 (8)	0 (0)	1 (8)	
Medication use					
Beta blocker, n (%)	0 (0)	1 (5)	1 (5)	0 (0)	0.56
Calcium channel blocker, n (%)	1 (5)	1 (5)	4 (21)	1 (5)	0.21
Antidyslipidemic medicine, n (%)	3 (15)	4 (20)	3 (16)	4 (21)	0.95
Hypoglycemic medicine, n (%)	0 (0)	1 (5)	0 (0)	0 (0)	0.40
Antianxiety agent, n (%)	1 (5)	0 (0)	0 (0)	0 (0)	0.40
Supplement compliance, %^†^	100.0 ± 0.0	99.6 ± 0.2	97.7 ± 0.9	96.4 ± 1.3*^⁑^	0.01
Participation rate in a weekly supervised session, %	–	–	92 ± 3	93 ± 5	0.91

Data are shown as the means ± standard error or frequency counts (%). *Placebo*, placebo without physical activity intervention; *LTP*, lactotripeptide without physical activity intervention; *placebo + PA*, placebo with physical activity intervention*; LTP + PA*, lactotripeptide with physical activity intervention. *P < 0.05 vs. placebo group; ^⁑^*P* < 0.05 vs. LTP group. ^†^Data available in 77 individuals.

The numbers of participants for whom SB, light-intensity PA (LPA), and MVPA could not be assessed during each study period are presented in Supplemental Table 1. The participants were asked to wear an accelerometer on their left hip during all study periods. To be eligible, participants had to wear the accelerometer for at least three days, with a total wear time of at least 10 h/day in each period of the study. If the data adoption criteria for SB, LPA, and MVPA were not met at each time point, the data were assumed to be missing. Several data points were missing for each study period, and the total data acquisition rate was approximately 95%. As shown in Table 2, the changes in LPA times did not significantly differ between groups during the intervention period. In contrast, a significant interaction was observed between changes in SB and MVPA times. In the placebo + PA and LTP + PA groups, the MVPA time was significantly longer at weeks 2–8 than at baseline.

**Table 2:** Time-related changes in the meantime spent in sedentary behavior, light-intensity physical activity, and moderate- to vigorous-intensity physical activity.

	Baseline	Week1	Week2	Week3	Week4	Week5	Week6	Week7	Week8		
Sedentary behavior, %											
Placebo	59.6 (54.8, 64.5)	61.6 (56.7, 66.5)	60.3 (55.5, 65.2)	59.4 (54.6, 64.3)	59.0 (54.0, 63.9)	58.9 (54.1, 63.8)	59.2 (54.4, 64.1)	60.4 (55.5, 65.2)	58.7 (53.8, 63.6)	Time:	*F* = 3.43, *P* = 0.001
LTP	58.0 (52.9, 63.2)	57.0 (51.8, 62.2)	59.4 (54.2, 64.5)	59.7 (54.6, 64.8)	58.8 (53.6, 63.9)	58.0 (52.9, 63.2)	56.5 (51.4, 61.7)	57.1 (52.0, 62.2)	58.4 (53.3, 63.6)	Group:	*F* = 3.30, *P* = 0.002
Placebo + PA	53.3 (48.4, 58.1)	50.7 (45.8, 55.6)*	51.8 (46.9, 56.7)	49.6 (44.7, 54.4)*^⁑^	50.1 (45.2, 54.9)	49.4 (44.5, 54.2)*	48.6 (43.8, 53.5)*^a^	47.1 (42.2, 52.0)*^⁑ac^	47.9 (43.0, 52.8)*^⁑a^	Interaction:	*F* = 1.60, *P* = 0.04
LTP + PA	52.2 (47.2, 57.2)	50.1 (45.1, 55.1)*	51.5 (46.5, 56.5)	51.8 (46.7, 56.8)	49.3 (44.3, 54.3)*	49.1 (44.1, 54.2)*	49.6 (44.6, 54.7)*	50.6 (45.5, 55.6)*	51.2 (46.2, 56.2)		
Light-intensity physical activity, %											
Placebo	34.9 (30.6, 39.2)	33.5 (29.2, 37.8)	34.4 (30.1, 38.7)	35.2 (30.9, 39.5)	35.0 (30.6, 39.4)	35.5 (31.2, 39.8)	35.3 (31.0, 39.6)	33.9 (29.6, 38.2)	35.6 (31.2, 39.9)	Time:	*F* = 1.09, *P* = 0.37
LTP	36.0 (31.4, 40.5)	37.0 (32.4, 41.6)	34.9 (30.4, 39.5)	34.8 (30.2, 39.3)	35.3 (30.7, 39.8)	35.8 (31.3, 40.4)	37.1 (32.5, 41.6)	36.3 (31.7, 40.8)	35.3 (30.8, 39.8)	Group:	*F* = 3.31, *P* = 0.03
Placebo + PA	41.6 (37.3, 45.9)	41.9 (37.6, 46.2)*	40.9 (36.5, 45.2)	42.6 (38.3, 46.9)	42.0 (37.7, 46.3)	42.5 (38.2, 46.8)	43.0 (38.7, 47.3)	43.9 (39.6, 48.2)*	44.0 (39.7, 48.3)*^⁑^	Interaction:	*F* = 1.17, *P* = 0.26
LTP + PA	41.3 (36.9, 45.7)	42.0 (37.6, 46.5)*	40.2 (35.8, 44.7)	39.6 (35.1, 44.1)	41.6 (37.2, 46.0)	41.7 (37.3, 46.1)	40.9 (36.4, 45.3)	40.2 (35.8, 44.6)	40.0 (35.5, 44.4)		
Moderate- to vigorous-intensity physical activity, %											
Placebo	5.4 (4.0, 6.8)	4.9 (3.6, 6.3)	5.3 (3.9, 6.7)	5.3 (3.9, 6.7)	6.0 (4.6, 7.5)	5.5 (4.1, 6.9)	5.5 (4.1, 6.9)	5.8 (4.4, 7.1)	5.7 (4.3, 7.1)	Time:	*F* = 12.47, *P* < 0.001
LTP	6.0 (4.5, 7.5)	6.0 (4.5, 7.5)	5.7 (4.2, 7.2)	5.5 (4.1, 7.0)	6.0 (4.5, 7.4)	6.2 (4.7, 7.6)	6.4 (4.9, 7.9)	6.6 (5.2, 8.1)	6.2 (4.8, 7.7)	Group:	*F* = 4.87, *P* = 0.004
Placebo + PA	5.1 (3.8, 6.5)	7.4 (6.0, 8.8)^a^	7.4 (6.0, 8.8)^a^	7.8 (6.4, 9.2)^a^	7.9 (6.5, 9.3)^a^	8.2 (6.8, 9.6)^a^	8.4 (7.1, 9.8)*^a^	9.0 (7.6, 10.4)*^abc^	8.1 (6.7, 9.5)^a^	Interaction:	*F* = 2.84, *P* < 0.001
LTP + PA	6.5 (5.1, 7.9)	7.9 (6.4, 9.3)*	8.2 (6.8, 9.7)*^a^	8.6 (7.2, 10.1)*^⁑a^	9.1 (7.7, 10.5)*^⁑a^	9.2 (7.7, 10.6)*^⁑a^	9.5 (8.1, 10.9)*^⁑ab^	9.2 (7.8, 10.7)*^a^	8.9 (7.4, 10.3)*^a^		

The number of participants for whom physical activity could not be assessed during each study period is shown in Supplementary Table 1. Data are presented as mean (95% confidence interval). *Placebo* placebo without physical activity intervention, *LTP* lactotripeptide without physical activity intervention, *placebo + PA* placebo with physical activity intervention, *LTP + PA* lactotripeptide with physical activity intervention. ^a^*P* < 0.05 vs. baseline (same group); ^b^*P* < 0.05 vs. week 1 (same group); ^c^*P* < 0.05 vs. week 2 (same group). ^⁎^*P* < 0.05 vs. placebo group (same study period); ^⁑^*P* < 0.05 vs. LTP group (same study period).

Table 3 shows the effects of LTP ingestion and PA intervention on anthropometric measurements, blood pressure, and dietary habits. Although LTP ingestion showed a significant main effect on weight and body fat, there were no significant interactions or main effects of LTP ingestion or PA intervention on body mass index, skeletal muscle mass, skeletal muscle mass index, or blood pressure. Total energy, dietary protein, fat, carbohydrate intake, and protein intake/body weight showed no significant interactions or main effects between LTP ingestion and PA intervention.

**Table 3:** Effects of LTP ingestion and physical activity intervention on anthropometry, blood pressure, and dietary habits (n = 78).

	Placebo (n = 20)	LTP (n = 20)	Placebo + PA (n = 19)	LTP + PA (n = 19)		
Weight, kg						
Baseline	55.6 ± 2.0	59.1 ± 1.9	59.0 ± 2.5	60.0 ± 2.3	LTP	*F* = 5.973, *P* = 0.017, partial *η*^2^ = 0.076
Post-intervention	55.2 ± 2.0	59.3 ± 1.9	58.6 ± 2.5	59.9 ± 2.2	Physical activity intervention	*F* = 0.234, *P* = 0.630, partial *η*^2^ = 0.003
Change	− 0.39 (− 0.69, − 0.10)	0.15 (− 0.33, 0.62)	− 0.44 (− 0.82, − 0.06)	− 0.09 (− 0.63, 0.44)	Interaction	*F* = 0.380, *P* = 0.539, partial *η*^2^ = 0.005
Body mass index, kg/m^2^						
Baseline	22.1 ± 0.6	23.6 ± 0.7	23.4 ± 0.8	23.1 ± 0.7	LTP	*F* = 3.258, *P* = 0.075, partial *η*^2^ = 0.043
Post-intervention	22.0 ± 0.6	23.6 ± 0.7	23.3 ± 0.8	23.0 ± 0.7	Physical activity intervention	*F* = 0.717, *P* = 0.400, partial *η*^2^ = 0.010
Change	− 0.12 (− 0.23, − 0.002)	0.07 (− 0.14, 0.27)	− 0.13 (− 0.25, − 0.01)	− 0.08 (− 0.28, 0.12)	Interaction	*F* = 1.532, *P* = 0.220, partial *η*^2^ = 0.021
Body fat, %						
Baseline	27.03 ± 1.86	28.28 ± 1.61	27.48 ± 1.45	28.76 ± 1.65	LTP	*F* = 4.086, *P* = 0.047, partial *η*^2^ = 0.053
Post-intervention	28.05 ± 1.93	29.39 ± 1.65	27.90 ± 1.54	29.11 ± 1.58	Physical activity intervention	*F* = 0.002, *P* = 0.968, partial *η*^2^ = 0.00002
Change	1.02 (0.29, 1.74)	1.11 (0.52, 1.7)	0.42 (− 0.12, 0.97)	0.35 (− 0.53, 1.23)	Interaction	*F* = 0.060, *P* = 0.807, partial *η*^2^ = 0.001
Skeletal muscle mass, kg						
Baseline	21.74 ± 0.91	22.78 ± 0.77	23.13 ± 1.06	23.01 ± 0.98	LTP	*F* = 2.273, *P* = 0.136, partial *η*^2^ = 0.030
Post-intervention	21.26 ± 0.88	22.51 ± 0.78	22.85 ± 1.09	22.86 ± 0.93	Physical activity intervention	*F* = 2.359, *P* = 0.129, partial *η*^2^ = 0.031
Change	− 0.49 (− 0.71, − 0.26)	− 0.27 (− 0.42, − 0.11)	− 0.27 (− 0.52, − 0.03)	− 0.15 (− 0.48, 0.19)	Interaction	*F* = 0.231, *P* = 0.632, partial *η*^2^ = 0.003
Skeletal muscle mass index, kg/m^2^						
Baseline	5.85 ± 0.12	6.14 ± 0.16	6.19 ± 0.18	5.95 ± 0.14	LTP	*F* = 1.896, *P* = 0.173, partial *η*^2^ = 0.025
Post-intervention	5.73 ± 0.12	6.05 ± 0.16	6.10 ± 0.18	5.89 ± 0.13	Physical activity intervention	*F* = 1.930, *P* = 0.169, partial *η*^2^ = 0.026
Change	− 0.12 (− 0.18, − 0.07)	− 0.09 (− 0.14, − 0.04)	− 0.09 (− 0.15, − 0.04)	− 0.05 (− 0.11, 0.005)	Interaction	*F* = 0.031, *P* = 0.860, partial *η*^2^ = 0.0004
Systolic blood pressure, mmHg						
Baseline	120.5 ± 3.7	122.4 ± 3.3	122.8 ± 3.2	128.2 ± 4.9	LTP	*F* = 0.0002, *P* = 0.989, partial *η*^2^ = 0.000003
Post-intervention	120.0 ± 3.6	120.7 ± 3.1	123.7 ± 3.7	129.3 ± 4.4	Physical activity intervention	*F* = 2.477, *P* = 0.120, partial *η*^2^ = 0.033
Change	− 0.54 (− 3.60, 2.52)	− 1.68 (− 4.86, 1.49)	0.94 (− 2.27, 4.14)	1.11 (− 3.70, 5.92)	Interaction	*F* = 0.277, *P* = 0.601, partial *η*^2^ = 0.004
Diastolic blood pressure, mmHg						
Baseline	73.7 ± 1.7	76.6 ± 2.5	75.1 ± 1.4	77.5 ± 3.3	LTP	*F* = 0.096, *P* = 0.757, partial *η*^2^ = 0.001
Post-intervention	74.2 ± 1.7	75.3 ± 2.4	75.5 ± 1.5	78.5 ± 3.3	Physical activity intervention	*F* = 1.366, *P* = 0.246, partial *η*^2^ = 0.018
Change	0.55 (− 1.10, 2.19)	− 1.29 (− 3.67, 1.09)	0.42 (− 1.66, 2.50)	1.01 (− 1.73, 3.75)	Interaction	*F* = 1.281, *P* = 0.261, partial *η*^2^ = 0.017
Pulse pressure, mmHg						
Baseline	46.9 ± 2.7	45.9 ± 1.4	47.6 ± 2.2	50.7 ± 2.4	LTP	*F* = 0.046, *P* = 0.831, partial *η*^2^ = 0.001
Post-intervention	45.8 ± 2.7	45.5 ± 1.5	48.2 ± 2.7	50.8 ± 2.1	Physical activity intervention	*F* = 1.293, *P* = 0.259, partial *η*^2^ = 0.017
Change	− 1.09 (− 3.38, 1.20)	− 0.40 (− 3.12, 2.32)	0.52 (− 1.68, 2.72)	0.10 (− 2.80, 3.01)	Interaction	*F* = 0.071, *P* = 0.790, partial *η*^2^ = 0.001
Heart rate, beats/min						
Baseline	58.7 ± 1.3	59.1 ± 1.2	61.1 ± 1.9	60.1 ± 2.1	LTP	*F* = 0.715, *P* = 0.401, partial *η*^2^ = 0.010
Post-intervention	57.6 ± 1.8	59.2 ± 1.4	59.0 ± 1.7	58.5 ± 2.2	Physical activity intervention	*F* = 1.461, *P* = 0.231, partial *η*^2^ = 0.020
Change	− 1.14 (− 3.45, 1.17)	0.09 (− 1.85, 2.02)	− 2.16 (− 4.22, − 0.11)	− 1.64 (− 3.75, 0.46)	Interaction	*F* = 0.178, *P* = 0.675, partial *η*^2^ = 0.002
Total energy, g/day						
Baseline	1682 ± 73	2018 ± 163	2003 ± 156	1864 ± 78	LTP	*F* = 0.768, *P* = 0.364, partial *η*^2^ = 0.010
Post-intervention	1734 ± 92	1961 ± 140	1860 ± 151	1925 ± 99	Physical activity intervention	*F* = 0.020, *P* = 0.888, partial *η*^2^ = 0.0002
Change	52 (− 135, 240)	− 57 (− 235, 120)	− 144 (− 370, 83)	61 (− 157, 279)	Interaction	*F* = 0.818, *P* = 0.369, partial *η*^2^ = 0.011
Protein intake, g/day						
Baseline	68.7 ± 4.2	86.5 ± 7.8	83.1 ± 7.4	75.9 ± 4.1	LTP	*F* = 0.003, *P* = 0.954, partial *η*^2^ = 0.00004
Post-intervention	72.7 ± 5.3	81.1 ± 7.2	77.1 ± 7.3	76.1 ± 4.3	Physical activity intervention	*F* = 0.163, *P* = 0.688, partial *η*^2^ = 0.002
Change	4.06 (− 5.15, 13.27)	− 5.40 (− 15.02, 4.22)	− 5.96 (− 15.15, 3.22)	0.20 (− 9.68, 10.08)	Interaction	*F* = 1.073, *P* = 0.304, partial *η*^2^ = 0.014
Protein intake/body weight, g/day kg						
Baseline	1.26 ± 0.09	1.52 ± 0.16	1.45 ± 0.15	1.31 ± 0.11	LTP	*F* = 0.120, *P* = 0.730, partial *η*^2^ = 0.002
Post-intervention	1.34 ± 0.10	1.44 ± 0.17	1.35 ± 0.14	1.29 ± 0.08	Physical activity intervention	*F* = 0.593, *P* = 0.444, partial *η*^2^ = 0.008
Change	0.08 (− 0.11, 0.26)	− 0.08 (− 0.25, 0.09)	− 0.10 (− 0.27, 0.07)	− 0.02 (− 0.19, 0.14)	Interaction	*F* = 0.999, *P* = 0.321, partial *η*^2^ = 0.013
Fat intake, g/day						
Baseline	56.3 ± 3.8	64.4 ± 4.6	62.6 ± 5.5	60.1 ± 3.8	LTP	*F* = 0.041, *P* = 0.840, partial *η*^2^ = 0.001
Post-intervention	55.5 ± 3.8	59.3 ± 3.4	58.2 ± 4.6	58.3 ± 3.4	Physical activity intervention	*F* = 0.017, *P* = 0.898, partial *η*^2^ = 0.0002
Change	− 0.77 (− 9.11, 7.58)	− 5.13 (− 13.66, 3.40)	− 4.37 (− 12.09, 3.36)	− 1.80 (− 11.74, 8.15)	Interaction	*F* = 0.022, *P* = 0.883, partial *η*^2^ = 0.0002
Carbohydrate intake, g/day						
Baseline	211.3 ± 10.5	252.1 ± 25.1	253.7 ± 24.9	232.5 ± 11.0	LTP	*F* = 1.570, *P* = 0.214, partial *η*^2^ = 0.021
Post-intervention	222.9 ± 12.5	260.3 ± 22.7	231.3 ± 22.7	239.0 ± 13.9	Physical activity intervention	*F* = 1.424, *P* = 0.237, partial *η*^2^ = 0.019
Change	11.59 (− 15.69, 38.87)	8.25 (− 12.32, 28.83)	− 22.43 (− 58.97, 14.12)	6.50 (− 19.4, 32.4)	Interaction	*F* = 0.368, *P* = 0.546, partial *η*^2^ = 0.005

Data are mean ± standard error for baseline and post-intervention and mean (95% confidence interval) for changes at 8 weeks. *Placebo* placebo without physical activity intervention; , *LTP* lactotripeptide without physical activity intervention, *placebo + PA* placebo with physical activity intervention, *LTP + PA* lactotripeptide with physical activity intervention.

The results of the simple correlations between fatigue and mood status before and after the intervention are shown in Supplemental Table 2. All indicators, except the visual analog scale, showed significant positive associations before and after the intervention.

Table 4 shows the effects of LTP ingestion and PA interventions on fatigue and mood status. No significant interactions or main effects of LTP ingestion or PA intervention were observed for any fatigue scale. In contrast, analyses of the main effects showed that the PA intervention significantly decreased the total mood disturbance (TMD) score of the Profile of Mood States second edition (POMS2) and the Beck Depression Inventory Second Edition (BDI-II) score. After post-hoc paired comparisons, a significant difference was found in the change in TMD and BDI-II scores between the LTP and LTP + PA groups, whereas no difference was found between the placebo and placebo + PA groups.

**Table 4:** Effects of LTP ingestion and physical activity intervention on fatigue and mood status (n = 78).

	Placebo (n = 20)	LTP (n = 20)	Placebo + PA (n = 19)	LTP + PA (n = 19)		
Visual analog scale, cm						
Baseline	2.90 ± 0.46	2.91 ± 0.40	2.93 ± 0.45	2.89 ± 0.42	LTP	*F* = 0.040, *P* = 0.842, partial *η*^2^ = 0.001
Post-intervention	3.05 ± 0.46	2.84 ± 0.51	2.23 ± 0.47	2.25 ± 0.45	Physical activity intervention	*F* = 2.193, *P* = 0.143, partial *η*^2^ = 0.029
Change	0.15 (− 1.16, 1.46)	− 0.08 (− 1.50, 1.35)	− 0.71 (− 2.20, 0.79)	− 0.64 (− 1.47, 0.18)	Interaction	*F* = 0.064, *P* = 0.802, partial *η*^2^ = 0.001
Brief Fatigue Inventory, points						
Baseline	2.10 ± 0.34	2.56 ± 0.38	2.29 ± 0.39	1.92 ± 0.32	LTP	*F* = 0.032, *P* = 0.859, partial *η*^2^ = 0.0004
Post-intervention	1.80 ± 0.38	2.00 ± 0.40	1.89 ± 0.41	1.63 ± 0.29	Physical activity intervention	*F* = 0.001, *P* = 0.972, partial *η*^2^ = 0.00001
Change	− 0.31 (− 1.17, 0.56)	− 0.56 (− 1.29, 0.17)	− 0.40 (− 0.99, 0.18)	− 0.30 (− 0.98, 0.38)	Interaction	*F* = 0.0002, *P* = 0.987, partial *η*^2^ = 0.000004
POMS2-AH (anger-hostility), points						
Baseline	43.8 ± 1.2	45.5 ± 1.2	45.3 ± 1.6	46.3 ± 1.5	LTP	*F* = 0.011, *P* = 0.919, partial *η*^2^ = 0.0001
Post-intervention	43.3 ± 1.1	44.3 ± 1.2	43.9 ± 1.3	44.6 ± 1.2	Physical activity intervention	*F* = 0.028, *P* = 0.867, partial *η*^2^ = 0.0004
Change	− 0.55 (− 2.51, 1.41)	− 1.15 (− 3.19, 0.89)	− 1.32 (− 3.12, 0.49)	− 1.68 (− 4.76, 1.39)	Interaction	*F* = 0.001, *P* = 0.977, partial *η*^2^ = 0.00001
POMS2-CB (confusion-bewilderment), points						
Baseline	42.5 ± 1.0	45.1 ± 1.3	44.7 ± 1.9	45.3 ± 1.2	LTP	*F* = 0.912, *P* = 0.343, partial *η*^2^ = 0.012
Post-intervention	45.3 ± 1.3	45.5 ± 1.6	44.8 ± 1.8	44.4 ± 1.3	Physical activity intervention	*F* = 1.623, *P* = 0.207, partial *η*^2^ = 0.022
Change	2.80 (0.45, 5.15)	0.40 (− 2.66, 3.46)	0.16 (− 2.07, 2.39)	− 0.89 (− 4.05, 2.26)	Interaction	*F* = 0.070, *P* = 0.792, partial *η*^2^ = 0.001
POMS2-DD (depression-dejection), points						
Baseline	44.9 ± 1.3	45.0 ± 0.8	45.5 ± 1.8	47.3 ± 1.1	LTP	*F* = 0.109, *P* = 0.743, partial *η*^2^ = 0.001
Post-intervention	44.6 ± 1.0	45.3 ± 0.9	44.8 ± 1.4	44.8 ± 0.9	Physical activity intervention	*F* = 2.075, *P* = 0.154, partial *η*^2^ = 0.028
Change	− 0.30 (− 1.71, 1.11)	0.30 (− 1.51, 2.11)	− 0.68 (− 2.44, 1.08)	− 2.42 (− 4.42, − 0.42)	Interaction	*F* = 1.579, *P* = 0.213, partial *η*^2^ = 0.021
POMS2-FI (fatigue-inertia), point						
Baseline	43.4 ± 1.0	43.1 ± 1.1	43.5 ± 1.8	44.1 ± 1.1	LTP	*F* = 0.106, *P* = 0.745, partial *η*^2^ = 0.001
Post-intervention	42.8 ± 1.0	42.9 ± 1.5	42.8 ± 1.9	42.3 ± 1.1	Physical activity intervention	*F* = 0.736, *P* = 0.394, partial *η*^2^ = 0.010
Change	− 0.60 (− 2.77, 1.57)	− 0.15 (− 2.28, 1.98)	− 0.74 (− 2.28, 0.81)	− 1.84 (− 3.98, 0.29)	Interaction	*F* = 0.543, *P* = 0.463, partial *η*^2^ = 0.007
POMS2-TA (tension-anxiety), points						
Baseline	43.4 ± 1.2	45.2 ± 1.2	47.2 ± 2.4	46.7 ± 1.8	LTP	*F* = 0.012, *P* = 0.912, partial *η*^2^ = 0.0001
Post-intervention	45.4 ± 1.2	45.3 ± 1.3	44.3 ± 1.5	44.8 ± 1.9	Physical activity intervention	*F* = 3.832, *P* = 0.054, partial *η*^2^ = 0.050
Change	2.00 (− 0.29, 4.29)	0.15 (− 2.67, 2.97)	− 2.89 (− 6.43, 0.64)	− 1.89 (− 4.64, 0.85)	Interaction	*F* = 0.639, *P* = 0.427, partial *η*^2^ = 0.009
POMS2-VA (vigor-activity), points						
Baseline	50.4 ± 1.6	51.7 ± 1.9	52.5 ± 3.3	53.5 ± 2.0	LTP	*F* = 0.080, *P* = 0.779, partial *η*^2^ = 0.001
Post-intervention	54.0 ± 1.7	53.6 ± 2.3	54.9 ± 2.4	57.8 ± 2.6	Physical activity intervention	*F* = 0.556, *P* = 0.458, partial *η*^2^ = 0.008
Change	3.65 (1.11, 6.19)	1.90 (− 2.62, 6.42)	2.42 (− 2.92, 7.76)	4.32 (1.15, 7.48)	Interaction	*F* = 1.007, *P* = 0.319, partial *η*^2^ = 0.014
POMS2-F (friendliness), points						
Baseline	49.3 ± 1.7	52.6 ± 2.0	54.8 ± 2.4	54.7 ± 1.9	LTP	*F* = 1.195, *P* = 0.278, partial *η*^2^ = 0.016
Post-intervention	52.9 ± 2.0	51.9 ± 2.2	56.4 ± 2.2	55.7 ± 3.0	Physical activity intervention	*F* = 0.180, *P* = 0.672, partial *η*^2^ = 0.002
Change	3.55 (− 0.01, 7.11)	− 0.70 (− 5.40, 4.00)	1.53 (− 1.97, 5.03)	1.00 (− 2.79, 4.79)	Interaction	*F* = 0.612, *P* = 0.436, partial *η*^2^ = 0.008
POMS2-TMD (total mood disturbance) score, points						
Baseline	43.1 ± 1.0	44.1 ± 0.9	43.9 ± 1.6	45.2 ± 1.3	LTP	*F* = 1.058, *P* = 0.307, partial *η*^2^ = 0.014
Post-intervention	43.2 ± 1.1	43.9 ± 1.0	42.9 ± 1.4	42.5 ± 1.0	Physical activity intervention	*F* = 5.216, *P* = 0.025, partial *η*^2^ = 0.067
Change	0.15 (− 1.26, 1.56)	− 0.20 (− 1.83, 1.43)	− 1.05 (− 2.54, 0.43)	− 2.74 (− 4.60, − 0.88)^⁑^	Interaction	*F* = 0.822, *P* = 0.368, partial *η*^2^ = 0.011
BDI-II, points						
Baseline	7.75 ± 1.46	6.50 ± 1.10	9.42 ± 1.54	8.79 ± 1.14	LTP	*F* = 0.018, *P* = 0.894, partial *η*^2^ = 0.0002
Post-intervention	7.20 ± 1.66	7.10 ± 1.35	7.89 ± 1.53	6.16 ± 0.91	Physical activity intervention	*F* = 4.807, *P* = 0.032, partial *η*^2^ = 0.062
Change	− 0.55 (− 1.39, 0.29)	0.60 (− 0.87, 2.07)	− 1.53 (− 3.53, 0.48)	− 2.63 (− 5.04, − 0.23)^⁑^	Interaction	*F* = 1.746, *P* = 0.190, partial *η*^2^ = 0.023

Data are presented as mean ± standard error for baseline and post-intervention and mean (95% confidence interval) for changes at 8 weeks. *Placebo *placebo without physical activity intervention, *LTP* lactotripeptide without physical activity intervention, *placebo + PA* placebo with physical activity intervention, *LTP + PA* lactotripeptide with physical activity intervention, *POMS2* Profile of Mood States second edition, *BDI-II* Beck Depression Inventory second edition. ^⁑^*P* < 0.05 vs. LTP group.

Although we confirmed that the participants were not taking angiotensin-converting enzyme inhibitors or angiotensin II receptor blockers before the study began, two participants in the placebo and LTP + PA groups were taking angiotensin II receptor blockers when their prescription records were checked after the study was completed. In addition, one participant in the LTP + PA group did not participate in any supervised session other than the first session, and no PA data were obtained. The results, excluding these three participants (taking angiotensin II receptor blockers, n = 2; rarely participated in supervised sessions, n = 1), are shown in Supplemental Table 3. There was still a significant main effect of the PA intervention on TMD and BDI-II scores.

**Figure 1:**
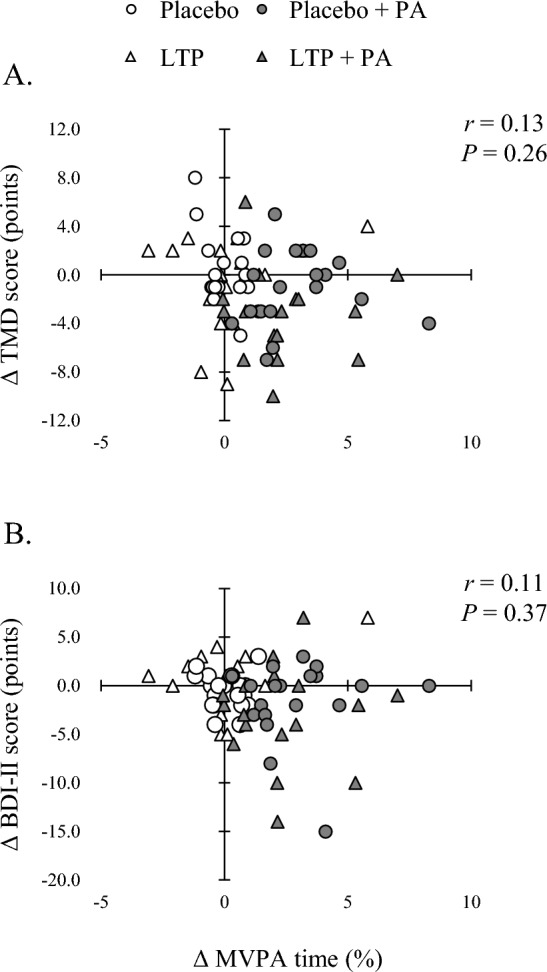
The simple correlations between the changes in MVPA time and the changes in TMD (**A**) and BDI-II (**B**) scores. The change in MVPA time was calculated by subtracting the baseline MVPA time from the average MVPA time during the intervention period. Five participants were unable to assess baseline MVPA time, so the analysis was performed with 73 participants. *Placebo* placebo without physical activity intervention, *LTP* lactotripeptide without PA intervention, *placebo + PA* placebo with PA intervention, *LTP + PA* lactotripeptide with PA intervention, *TMD* total mood disturbance, *BDI*-*II* Beck Depression Inventory second edition.

Figure 1 shows the simple correlations between the changes in MVPA time and TMD and BDI-II scores. The baseline MVPA time could not be assessed in five participants; therefore, the analysis was performed with 73 participants. The change in MVPA time was calculated by subtracting the baseline MVPA time from the average MVPA time during the intervention period. There was no significant association between changes in MVPA time and changes in TMD or BDI-II scores.

## Discussion

This study investigated the combined effects of LTP ingestion and PA interventions on fatigue status. There were no dropouts and all randomized participants completed the study. Supplement compliance was considerably higher in all groups (> 95%). The time spent on PA was evaluated as > 95% of the data in the study period, and the MVPA time in the placebo + PA and LTP + PA groups was significantly higher during the intervention period than at baseline. Therefore, the intervention was appropriately implemented. In this study, no significant interactions or main effects of LTP ingestion and PA intervention were observed on any of the fatigue scales. In contrast, the PA intervention improved TMD and BDI-II scores. After post-hoc paired comparisons, a significant difference was noted in the change in TMD and BDI-II scores between the LTP and LTP + PA groups, whereas no difference was found between the placebo and placebo + PA groups. These results suggest that PA intervention improves mood status and depressive symptoms, and that LTP ingestion may enhance its effectiveness.

In recent years, patient-reported outcomes have been emphasized to facilitate the transition from normal to successful aging^21–26^. Fatigue and mood status play various roles in psychological well-being and psychopathology^27^. Mood disorders are common in older adults and can be associated with numerous problems such as reduced psychological functioning, increased depression and anxiety, and poor life satisfaction^27,28^. Although several pharmacological therapies are available, they have potential side effects^29^. Therefore, establishing safe therapies to improve fatigue and mood status is clinically important in middle-aged and older adults. Our results showed that LTP ingestion and PA intervention did not affect fatigue status. However, PA intervention improves mood status and depressive symptoms and LTP ingestion may enhance its effectiveness. Our findings may contribute to the establishment of intervention programs to address psychological problems in middle-aged and older adults.

Both psychological and physiological mechanisms, such as the distraction and monoamine hypotheses, have been shown to have beneficial effects on mood and emotional well-being^30^. Thus, it appears that the PA intervention improved TMD and BDI-II scores. In contrast, previous studies reported a significant association between vital exhaustion and vascular function^12,13^. In addition, interventions that combine increased PA and nutritional supplementation may have additional effects on quality of life and depressive symptoms^19,20^. Based on these reports, we hypothesized that PA intervention combined with LTP ingestion may be additive in improving the fatigue status, which is greater than that observed with either treatment alone. In this study, the main effect of LTP ingestion was not significant for any outcome, but LTP ingestion enhanced the effect of the PA intervention on improving mood status and depressive symptoms. This was an exploratory randomized controlled trial to examine the combined effects of LTP ingestion and PA intervention and is worthy of further confirmatory trials.

Analyses of the main effects showed that the PA intervention significantly decreased TMD and BDI-II scores. However, there was no significant association between changes in MVPA time and changes in TMD or BDI-II scores. There may be a ceiling effect on the ability of increased MVPA to improve mood status and depressive symptoms. Among the previous studies that have investigated the effects of PA interventions on fatigue and mood status, none have assessed SB, LPA, and MVPA times during the intervention period as continuously as this study did^19,20^. Further research is required to test the hypotheses derived in this study.

Several studies have shown that middle-aged and older adults are more likely to suffer from malnutrition and depression, both of which can have negative consequences on overall health and well-being^31^. However, individual diets vary, making it difficult to determine the precise source of diets that cause mental health problems^32^. On the other hand, LTP is an easily available nutritional supplement and has also been shown to be safe in case of overdose^7,8^. Although there were no significant main effects of LTP ingestion on the fatigue scales, our results showed that LTP ingestion may enhance the effects of PA intervention on improving mood status and depressive symptoms. LTP ingestion may be an effective adjunct therapy for middle-aged and older adults to improve mood status and depressive symptoms, even if their dietary habits are difficult to improve.

In the present study, there were no changes in any of the fatigue scales before or after the intervention. We used a self-report questionnaire to assess the fatigue status. Objective measures of fatigue status, such as blood and salivary biomarkers, may also require assessment^33^. Additionally, the participants were healthy middle-aged and older adults recruited through local newspaper advertisements, and their fatigue status was mild at baseline, regardless of the questionnaire used to survey them. Patients with chronic fatigue syndrome or cancer, who exhibit a more severe fatigue status than the present participants, may have different results from those of the present study.

The time spent in MVPA significantly increased in the placebo + PA and LTP + PA groups during the intervention period. In the post-hoc test, MVPA time was significantly higher in the LTP + PA group than in the placebo group from weeks 1 to 8. In contrast, at weeks 6–7, MVPA time was higher in the placebo + PA group than in the placebo group. Furthermore, MVPA time in the LTP group did not differ from that in the placebo + PA group during the entire study period. A previous study suggested that mood disorder scores are lower in active than in inactive older women^17^. This report suggests that mood disorders are a cause of physical inactivity. Therefore, the present results suggest that LTP ingestion may enhance the effect of PA intervention on improving mood status and depressive symptoms, and indirectly contribute to increasing MVPA time.

SB is defined as any waking behavior with an energy expenditure ≤ 1.5 metabolic equivalents (METs) while sitting, reclining, or lying^34^. Growing evidence has shown that excessive SB may increase the risk of several chronic diseases, including cardiovascular disease, cancers, and type 2 diabetes, and decrease the quality of life^35,36^. In the present study, there were significant group differences in changes in SB time during the intervention period, although the participants were not educated about SB. An intervention involving SB in addition to MVPA could further enhance improvements in mood status and depressive symptoms.

A previous study reported that LTP inhibits angiotensin-converting enzymes^7^. Increased PA has also been shown to lower blood pressure^37^. However, in the present study, the blood pressure did not change after the intervention. Ishida et al. reported that the anti-hypertensive effect of LTP ingestion was not observed in normotensive individuals^8^. In the present study, most participants were normotensive; therefore, the antihypertensive effects of LTP ingestion and PA intervention may not have been observed.

This study has several strengths. First, this was a randomized controlled trial that assessed the combined effects of LTP ingestion and PA intervention. Second, a double-blind placebo-controlled design was used to test the effects of LTP ingestion. Finally, supplement compliance and time-related changes in PA were evaluated in detail. However, this study has several limitations. First, the number of male participants was relatively small. Second, two participants were taking angiotensin II receptor blockers, which violated the inclusion criteria. Third, although the participants were of a relatively broad age range, it was not possible to stratify and randomize them by age. Fourth, it is possible that responding to the questionnaires induced fatigue because the participants were asked to complete multiple questionnaires in a single day following an overnight fast of more than 12 h. Fifth, a control group that did not participate in either the supplement or PA intervention could have expanded the interpretation of this study. Finally, as this study was an exploratory randomized controlled trial with a small sample size, further confirmatory trials should be conducted in the future.

This study suggests that eight weeks of LTP ingestion and PA intervention did not have a significant effect on fatigue status. However, PA interventions improve mood status and depressive symptoms, and the ingestion of LTP may enhance their effectiveness. Our findings may contribute to the establishment of intervention programs to address psychological problems in middle-aged and older adults.

## Methods

### Study design and participants

The protocol was registered in the University Hospital Medical Information Network (UMIN) Clinical Trials Registry (UMIN000044896; registered on 18/7/2021). This 8-week randomized controlled trial was conducted at the University of Tsukuba, Japan, between July and October 2021. The intervention period (8 weeks) was determined based on our previous studies that examined the combined effects of LTP ingestion and aerobic exercise^10,11,38^. Participants were recruited through local newspaper advertisements.

**Figure 2:**
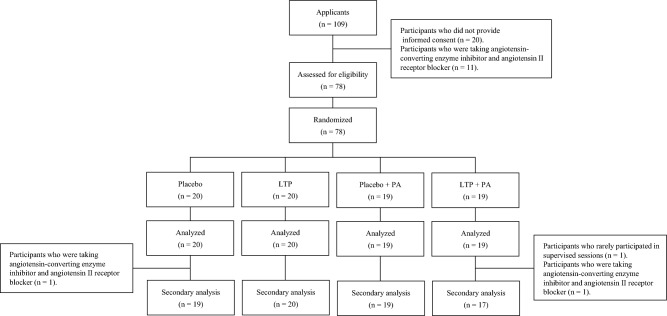
Flow diagram of the participant’s progress through the 8-week randomized trial. *Placebo* placebo without physical activity intervention, *LTP* lactotripeptide without PA intervention, *placebo + PA* placebo with PA intervention, *LTP + PA* lactotripeptide with PA intervention.

The inclusion criteria were as follows: (1) age ≥ 45 years, (2) not restricted by a doctor from exercising, (3) not performing moderate- to vigorous-intensity aerobic training, (4) not participating in another clinical trial within 1 year, (5) not having lactose intolerance, (6) agreeing with the contents of the study, and (7) not taking angiotensin-converting enzyme inhibitor or angiotensin II receptor blocker. The exclusion criteria were as follows: (1) care-dependent or support-dependent on the Japanese long-term care insurance system; (2) presence of severe heart disease, cerebrovascular disease, and renal dysfunction; (3) the patient’s medical doctor did not agree to their participation in the study; and (4) limited mobility. In total, 109 participants were contacted to participate in the study. Among them, 20 participants did not provide informed consent, and 11 were taking angiotensin-converting enzyme inhibitors or angiotensin II receptor blockers. Therefore, 78 middle-aged and older adults were enrolled in this interventional study (Fig. 2). This sample size could detect the effect size partial *η*^2^ = 0.20 in a priori power analysis for a repeated-measures analysis of variance (ANOVA) to test the interaction between within-subject factors and between-subject factors. As none of the participants met the exclusion criteria, 78 participants were randomly assigned to one of the following four groups using stratified randomization procedures involving computerized random numbers: placebo without PA intervention (placebo, n = 20), LTP without PA intervention (LTP, n = 20), placebo with PA intervention (placebo + PA, n = 19), or LTP with PA intervention (LTP + PA, n = 19). Owing to sex differences in fatigue status^39^, participants were stratified by sex when randomly grouped. Allocation data were generated by an investigator who had no contact with the participants and were maintained at a central secure location until completion of the study. LTP or placebo tablets were individually packaged as three tablets and distinguished by red or blue labels, with no indication of whether they were placebo or LTP. Other staff members received information from the allocation investigator about whether each participant would participate in the PA intervention and whether they would ingest red or blue tablets and made this information known to the participants.

This study was approved by the Ethics Committee of the University of Tsukuba, institute of Health and Sports Sciences (approval no. Tai 020-168). The study conformed to the principles outlined in the Declaration of Helsinki and all participants provided written informed consent.

### Lactotripeptide ingestion

A double-blind placebo-controlled design was used to test the effects of LTP ingestion. During the study, participants received either LTP or placebo tablets. As described previously^38^, LTP active products were prepared from casein hydrolysate containing VPP and IPP, and the placebo was prepared using sodium caseinate instead of casein hydrolysate. The placebo and LTP tablets had similar appearance, smell, and taste. The average weight of one tablet was 253.0 ± 5.0 mg. Participants ingested three tablets daily. Each tablet was individually packaged as three tablets. The three LTP tablets contained 1.4 mg VPP and 2.0 mg IPP. We defined the administered dose based on a previous study, which confirmed that a single dose of 1.4 mg VPP and 2.0 mg IPP reduced fatigue in middle-aged and older men^13^. VPP and IPP contents were determined by grinding the three tablets using LC–MS/MS. Participants were instructed to consume their daily dose in the morning, preferably at breakfast. The time at which they consumed the tablets was recorded in the supplement diary. Supplement compliance (%) was calculated using the following formula using the supplement diary: ([days of intervention – days not consumed – days not recorded in the supplement diary]/days of intervention) × 100. The participants were also instructed not to change their dietary habits or medication use.

### Physical activity intervention

Individuals in the placebo + PA and LTP + PA groups participated in the PA intervention. The PA intervention consisted of weekly supervised and home-based sessions.

During the supervised sessions, exercises were performed using a cycling ergometer (900U; COMBI WELLNESS, Japan). During exercise, the heart rate was monitored using the ear sensors on the cycling ergometer. Exercise intensities and durations are listed in Supplemental Table 4. The estimated maximal heart rate was calculated using the formula, 207 − 0.7 × age^40^. The participants pedaled a bicycle at 60 rpm with the exercise intensity adjusted by the pedal weight. If the target heart rate was reached but the subjective exercise intensity assessed by the Borg scale was less than the target scale^41^, the exercise intensity was increased. Before and after the bicycle exercise, about 15 min of preparatory and organizational exercises were conducted before and after bicycle exercises.

In a supervised session, we educated the participants on MVPA at home. Accelerometer data were used to provide weekly feedback on MVPA time and step counts. The feedback sheet also included a graph showing the change in MVPA time over the intervention period. The participants were instructed to aim progressively to achieve the goals listed in Supplemental Table 5. Once one goal was met twice (i.e., 2 weeks), the participants moved on to the next goal. The participants were instructed to walk or jog at home, which they found somewhat hard (i.e., the Bord scale 13)^41^, to achieve both the MVPA and step count goals. Participants were not educated on SB or LPA.

### Measurements

#### Procedure

Physical examinations were performed before and after the intervention. Before each test, the participants were requested to abstain from caffeine, alcohol, and strenuous PA for a minimum of 24 h. The participants arrived at the laboratory 12 h postprandially. Anthropometric measurements were obtained after the participants arrived at the laboratory. Subsequently, self-reported questionnaires were administered. Hemodynamic parameters were measured after sitting at rest for at least 20 min. All procedures were performed at ambient room temperature (24–26 °C). Medication use was assessed using prescription records.

#### Self-administered questionnaire

The participants were asked to complete the following four self-administered questionnaires: visual analog scale, Brief Fatigue Inventory (BFI), POMS2 (Kanekoshobo, Tokyo, Japan), and BDI-II (Nihon Bunka Kagakusha, Tokyo, Japan). The participants were asked to complete all questionnaires on the day of the laboratory measurements. Participants completed the questionnaire following an overnight fast of more than 12 h, as with the other measurements. The participants completed the questionnaires at their own pace, and there was no time limit. Questionnaires were administered in the same manner before and after the intervention. Using the visual analog scale, participants were asked to indicate the intensity of perceived fatigue on a 100-mm horizontal line. The left side stated, “having no fatigue”, whereas the right side stated, “having maximum fatigue”^13^. The left side was defined as 0 cm and evaluated in 0.1 cm increments. The BFI, a self-rating assessment composed of nine items using a numerical scale of 0–10, was developed for rapid assessment of fatigue severity^42,43^. It has been translated into Japanese, and its reliability and validity have been previously confirmed^44^. Severity was classified into three groups as follows: 1–3 points, mild symptoms; 4–6 points, moderate symptoms; and 7–10 points, severe symptoms. POMS2 is a psychological rating scale used to assess fatigue and mood status. The POMS2 has been translated into Japanese, and its reliability and validity have been previously confirmed^45^. The original version of POMS2 consists of 65 items. Participants were asked to indicate their mood states on a scale of 0 (not at all) to 4 (extremely) points during the previous 1-week period. The POMS2 evaluates seven domains (anger-hostility [AH], confusion-bewilderment [CB], depression-dejection [DD], fatigue-inertia [FI], tension-anxiety [TA], vigor-activity [VA], and friendliness [F]). The TMD score was calculated using the following formula: (AH + CB + DD + FI + TA) − VA. T-scores for the seven domains and TMD scores were used in the analyses^39^. Higher scores indicate more severe fatigue and mood status. The BDI-II is a widely used 21-item self-report inventory that measures depression severity in adolescents and adults^46^. A previous study supported the use of the Japanese version of the BDI-II as a reliable and valid measure of subjective depressive symptoms in clinical practice and research^47^. Each item was scored on a scale of 0–3, and the total score was calculated by summing the scores for all items. The standard cut-off scores were as follows: 10–16 points, mild depression; 17–29 points, moderate depression; and 30–63 points, severe depression.

#### Sedentary behavior and physical activity

In the present study, we educated the participants on MVPA at home. To assess changes in the time spent in SB, LPA, and MVPA at home, we used a triaxial accelerometer (Active style Pro HJA-750C; Omron Healthcare, Kyoto, Japan). The participants were asked to wear an accelerometer on their left hip during all study periods. SB, LPA, and MVPA times were analyzed at nine points (baseline and each week of the intervention period). The accelerometer estimated the intensity of each activity by METs using a built-in algorithm and summarized it using 1-min epochs^48^. SB, LPA, and MVPA were classified as follows: SB, ≤ 1.5 METs, LPA, 1.6–2.9 METs, and MVPA, ≥ 3.0 METs^49–52^. Non-wear time was defined as at least consecutive 60 min of no activity (≤ 0.9 METs), with allowance for up to 2 min of some limited movement within those periods^48^. To be eligible, participants had to wear the accelerometer for at least 3 days, with a total wear time of at least 10 h/day in each period of the study^49,53^. The time spent per day on each behavioral variable was calculated by averaging the eligible data. The SB, LPA, and MVPA times were significantly affected by total wear time^54^. In this study, SB, LPA, and MVPA times were analyzed at nine time points (baseline and each week of the intervention period). Because the total wear time was different at each of the nine points, it was difficult to statistically adjust the total wear time when analyzing the changes in SB, LPA, and MVPA times during the intervention period. Therefore, SB, LPA, and MVPA times were divided by the total wear time at each study period and used in the analysis. The accelerometers for the participants in the placebo and LTP groups showed only a clock to reduce the bias of being active while wearing the accelerometer^55^. The accelerometers for participants in the placebo + PA and LTP + PA groups showed the clock and number of steps per day. As the participants in the placebo + PA and LTP + PA groups exchanged accelerometers in a weekly supervised session to provide weekly feedback, data on the day of the weekly supervised session were excluded from the analysis. A previous study reported that the intensity of activities assessed using this accelerometer showed a linear relationship with METs, as calculated using indirect calorimetry at home and during locomotive activities^48^. Detailed information regarding the validity of this accelerometer has been described elsewhere^48,56,57^.

#### Anthropometric measurements

Anthropometric measurements were performed with each participant barefoot and wearing light clothing. The hydration status was not controlled when anthropometric measurements were performed. Height was measured by using a wall-mounted stadiometer (AD-6227; A&D, Tokyo, Japan). Body composition was analyzed by bioelectrical impedance using a tetrapolar eight-point tractile electrode system (InBody 770; InBody Japan, Tokyo, Japan). Body mass index was calculated based on height and weight. The skeletal muscle mass index was calculated according to previous studies^58^.

#### Blood pressure and heart rate

Using a previously described noninvasive vascular profiling system (Form PWV/ABI; Colin Medical Technology, Aichi, Japan), brachial systolic blood pressure (bSBP), brachial diastolic blood pressure (bDBP), and heart rate were measured thrice in the supine position^59^. The mean of the three measurements was used in the analysis.

#### Dietary assessment

Dietary intake was evaluated using a brief self-administered dietary history questionnaire (BDHQ). The BDHQ includes questions about general dietary behavior and major cooking methods, frequency and amount of intake of five alcoholic beverages, and frequency of consumption of 50 selected food and non-alcoholic beverage items. The reproducibility and validity of this questionnaire have been confirmed elsewhere^59,60^.

### Statistical analysis

Randomized controlled trials often suffer from two major complications (non-compliance and missing outcomes). To solve these problems, we applied a statistical concept called intention-to-treat (ITT) analysis to examine changes in outcomes in the four groups during the intervention periods^61^. Group comparisons of clinical characteristics were performed using one-way analysis of variance (ANOVA) for continuous variables and chi-square tests for categorical variables. Linear mixed-effects models were used to evaluate changes in SB, LPA, and MVPA times during the intervention period. In the case of a significant *F* value, a post hoc test using the Bonferroni method identified significant differences among the mean values. To detect interactions in mean changes of the outcomes between the two factors (LTP or placebo and PA intervention or control), before and after the intervention, we performed a two-way analysis of covariance (ANCOVA). We also tested the main effects of LTP ingestion and PA intervention. If a significant interaction or main effect was observed, post hoc paired comparisons were corrected using the Bonferroni method. The baseline values of the outcomes were adjusted using linear mixed-effects models and two-way ANCOVA. The present study described partial *η*^2^ as a measure of effect size. The partial *η*^2^ of 0.0099, 0.0588, and 0.1379 indicate small, medium, and large effects, respectively^62^. We used Pearson’s correlation coefficient (*r*) to assess the correlations between several indicators. Significance was set at *P* < 0.05. Data were analyzed using IBM SPSS Statistics for Windows, version 28.0 (IBM Japan, Tokyo, Japan).

### Ethics approval and consent to participate

All the participants provided written informed consent. This study was conducted in accordance with the Declaration of Helsinki and approved by the ethical committee of the University of Tsukuba (approval no. Tai020-168).

### Supplementary Information

Supplementary Tables.

## Data Availability

The authors confirm that the data supporting the findings of this study are available within the article.
