# Supplementary material for: Effects of lactotripeptide ingestion and physical activity intervention on the fatigue status of middle-aged and older adults: a randomized controlled trial
Source: Sci Rep. 2023 Sep 21;13:15736. doi: 10.1038/s41598-023-41669-2 (PMC10514187; doi:10.1038/s41598-023-41669-2)
Supplement: Supplementary file 1 — Supplementary Tables. [file 41598_2023_41669_MOESM1_ESM.docx]

|  | Baseline | Week1 | Week2 | Week3 | Week4 | Week5 | Week6 | Week7 | Week8 |  |
| --- | --- | --- | --- | --- | --- | --- | --- | --- | --- | --- |
| Placebo, n (%) | 1 (5) | 0 (0) | 0 (0) | 1 (5) | 4 (20) | 0 (0) | 0 (0) | 0 (0) | 2 (10) |  |
| LTP, n (%) | 3 (15) | 3 (15) | 1 (5) | 1 (5) | 1 (5) | 1 (5) | 1 (5) | 1 (5) | 1 (5) |  |
| Placebo + PA, n (%) | 0 (0) | 0 (0) | 2 (10) | 0 (0) | 0 (0) | 1 (5) | 0 (0) | 1 (5) | 0 (0) |  |
| LTP + PA, n (%) | 1 (5) | 2 (10) | 3 (15) | 4 (20) | 1 (5) | 2 (10) | 3 (15) | 2 (10) | 2 (10) |  |
| Supplemental Table 1. Number of participants for whom physical activity could not be assessed during each study period. Data are presented as numbers (%). Placebo, placebo without physical activity intervention; LTP, lactotripeptide without physical activity intervention; placebo + PA, placebo with physical activity intervention; LTP+PA, lactotripeptide with physical activity intervention. | | | | | | | | | | |

|  |  |  | After intervention | | | | | | | | | | | |
| --- | --- | --- | --- | --- | --- | --- | --- | --- | --- | --- | --- | --- | --- | --- |
|  |  |  | 1 | 2 | 3 | 4 | 5 | 6 | 7 | 8 | 9 | 10 | 11 |  |
| Before intervention | 1. Visual analogue scale | *r* | 0.076 | 0.108 | 0.325 | 0.106 | 0.275 | 0.268 | 0.130 | 0.054 | -0.102 | 0.234 | 0.277 |  |
|  |  | *P* | 0.511 | 0.345 | 0.004 | 0.356 | 0.015 | 0.018 | 0.257 | 0.637 | 0.372 | 0.040 | 0.014 |  |
|  | 2. Brief Fatigue Inventory | *r* |  | 0.557 | 0.211 | 0.156 | 0.333 | 0.450 | 0.461 | -0.046 | 0.002 | 0.387 | 0.282 |  |
|  |  | *P* |  | < 0.001 | 0.064 | 0.174 | 0.003 | < 0.001 | < 0.001 | 0.687 | 0.983 | < 0.001 | 0.012 |  |
|  | 3. POMS2-AH (Anger-Hostility) | *r* |  |  | 0.651 | 0.484 | 0.586 | 0.398 | 0.535 | -0.039 | 0.006 | 0.641 | 0.473 |  |
|  |  | *P* |  |  | < 0.001 | < 0.001 | < 0.001 | < 0.001 | < 0.001 | 0.735 | 0.959 | < 0.001 | < 0.001 |  |
|  | 4. POMS2-CB (Confusion-Bewilderment) | *r* |  |  |  | 0.580 | 0.561 | 0.374 | 0.468 | 0.036 | 0.039 | 0.536 | 0.438 |  |
|  |  | *P* |  |  |  | < 0.001 | < 0.001 | < 0.001 | < 0.001 | 0.752 | 0.731 | < 0.001 | < 0.001 |  |
|  | 5. POMS2-DD (Depression-Dejection) | *r* |  |  |  |  | 0.756 | 0.484 | 0.504 | -0.056 | -0.042 | 0.673 | 0.616 |  |
|  |  | *P* |  |  |  |  | < 0.001 | < 0.001 | < 0.001 | 0.626 | 0.717 | < 0.001 | < 0.001 |  |
|  | 6. POMS2-FI (Fatigue-Inertia) | *r* |  |  |  |  |  | 0.742 | 0.442 | -0.103 | -0.136 | 0.624 | 0.488 |  |
|  |  | *P* |  |  |  |  |  | < 0.001 | < 0.001 | 0.371 | 0.235 | < 0.001 | < 0.001 |  |
|  | 7. POMS2-TA (Tension-Anxiety) | *r* |  |  |  |  |  |  | 0.612 | 0.027 | 0.105 | 0.540 | 0.443 |  |
|  |  | *P* |  |  |  |  |  |  | < 0.001 | 0.817 | 0.359 | < 0.001 | < 0.001 |  |
|  | 8. POMS2-VA (Vigor-Activity) | *r* |  |  |  |  |  |  |  | 0.639 | 0.565 | -0.209 | -0.186 |  |
|  |  | *P* |  |  |  |  |  |  |  | < 0.001 | < 0.001 | 0.066 | 0.103 |  |
|  | 9. POMS2-F (Friendliness) | *r* |  |  |  |  |  |  |  |  | 0.646 | -0.238 | -0.247 |  |
|  |  | *P* |  |  |  |  |  |  |  |  | < 0.001 | 0.036 | 0.029 |  |
|  | 10. POMS2-TMD (total mood disturbance) | *r* |  |  |  |  |  |  |  |  |  | 0.774 | 0.640 |  |
|  |  | *P* |  |  |  |  |  |  |  |  |  | < 0.001 | < 0.001 |  |
|  | 11. BDI-Ⅱ | *r* |  |  |  |  |  |  |  |  |  |  | 0.797 |  |
|  |  | *P* |  |  |  |  |  |  |  |  |  |  | < 0.001 |  |
| Supplemental Table 2. Pearson’s correlation coefficients and (*P*-values) for fatigue and mood status before and after intervention (n = 78). POMS2, Profile of Mood States second edition; BDI-II, Beck Depression Inventory second edition. | | | | | | | | | | | | | | |

|  | Placebo | | |  | LTP | | |  | Placebo + PA | | |  | LTP + PA | | |  |  |  |  |
| --- | --- | --- | --- | --- | --- | --- | --- | --- | --- | --- | --- | --- | --- | --- | --- | --- | --- | --- | --- |
|  | (n = 19) | | |  | (n = 20) | | |  | (n = 19) | | |  | (n = 17) | | |  |  |  |  |
| Visual analog scale, cm |  |  |  |  |  |  |  |  |  |  |  |  |  |  |  |  |  |  |  |
| Baseline | 3.05 | ± | 0.46 |  | 2.91 | ± | 0.40 |  | 2.93 | ± | 0.45 |  | 2.95 | ± | 0.46 |  | LTP: | *F* = 0.063, *P* = 0.803, partial *η*^2^ = 0.001 |  |
| Post-intervention | 2.77 | ± | 0.39 |  | 2.84 | ± | 0.51 |  | 2.23 | ± | 0.47 |  | 2.38 | ± | 0.49 |  | Physical activity intervention: | *F* = 1.122, *P* = 0.293, partial *η*^2^ = 0.016 |  |
| Change | -0.28 (-1.28, 0.72) | | |  | -0.08 (-1.50, 1.35) | | |  | -0.71 (-2.20, 0.79) | | |  | -0.57 (-1.47, 0.33) | | |  | Interaction: | *F* = 0.004, *P* = 0.948, partial *η*^2^ = 0.00006 |  |
| Brief Fatigue Inventory, points |  |  |  |  |  |  |  |  |  |  |  |  |  |  |  |  |  |  |  |
| Baseline | 2.21 | ± | 0.33 |  | 2.56 | ± | 0.38 |  | 2.29 | ± | 0.39 |  | 2.02 | ± | 0.34 |  | LTP: | *F* = 0.010, *P* = 0.919, partial *η*^2^ = 0.0001 |  |
| Post-intervention | 1.59 | ± | 0.34 |  | 2.00 | ± | 0.40 |  | 1.89 | ± | 0.41 |  | 1.59 | ± | 0.32 |  | Physical activity intervention: | *F* = 0.104, *P* = 0.748, partial *η*^2^ = 0.001 |  |
| Change | -0.62 (-1.22, -0.01) | | |  | -0.56 (-1.29, 0.17) | | |  | -0.40 (-0.99, 0.18) | | |  | -0.43 (-1.15, 0.30) | | |  | Interaction: | *F* = 0.252, *P* = 0.617, partial *η*^2^ = 0.004 |  |
| POMS2-AH (Anger-Hostility), points |  |  |  |  |  |  |  |  |  |  |  |  |  |  |  |  |  |  |  |
| Baseline | 44.0 | ± | 1.3 |  | 45.5 | ± | 1.2 |  | 45.3 | ± | 1.6 |  | 46.8 | ± | 1.6 |  | LTP: | *F* = 0.038, *P* = 0.846, partial *η*^2^ = 0.001 |  |
| Post-intervention | 42.9 | ± | 1.1 |  | 44.3 | ± | 1.2 |  | 43.9 | ± | 1.3 |  | 44.1 | ± | 1.2 |  | Physical activity intervention: | *F* = 0.222, *P* = 0.639, partial *η*^2^ = 0.003 |  |
| Change | -1.05 (-2.8, 0.70) | | |  | -1.15 (-3.19, 0.89) | | |  | -1.32 (-3.12, 0.49) | | |  | -2.76 (-5.77, 0.24) | | |  | Interaction: | *F* = 0.567, *P* = 0.454, partial *η*^2^ = 0.008 |  |
| POMS2-CB (Confusion-Bewilderment), points |  |  |  |  |  |  |  |  |  |  |  |  |  |  |  |  |  |  |  |
| Baseline | 42.7 | ± | 1.0 |  | 45.1 | ± | 1.3 |  | 44.7 | ± | 1.9 |  | 45.2 | ± | 1.3 |  | LTP: | *F* = 0.855, *P* = 0.358, partial *η*^2^ = 0.012 |  |
| Post-intervention | 45.2 | ± | 1.4 |  | 45.5 | ± | 1.6 |  | 44.8 | ± | 1.8 |  | 44.1 | ± | 1.5 |  | Physical activity intervention: | *F* = 1.508, *P* = 0.223, partial *η*^2^ = 0.021 |  |
| Change | 2.42 (0.09, 4.76) | | |  | 0.40 (-2.66, 3.46) | | |  | 0.16 (-2.07, 2.39) | | |  | -1.12 (-4.59, 2.35) | | |  | Interaction: | *F* = 0.004, *P* = 0.953, partial *η*^2^ = 0.00005 |  |
| POMS2-DD (Depression-Dejection), points |  |  |  |  |  |  |  |  |  |  |  |  |  |  |  |  |  |  |  |
| Baseline | 45.1 | ± | 1.3 |  | 45.0 | ± | 0.8 |  | 45.5 | ± | 1.8 |  | 47.5 | ± | 1.2 |  | LTP: | *F* = 0.116, *P* = 0.734, partial *η*^2^ = 0.002 |  |
| Post-intervention | 44.6 | ± | 1.1 |  | 45.3 | ± | 0.9 |  | 44.8 | ± | 1.4 |  | 44.8 | ± | 1.0 |  | Physical activity intervention: | *F* = 2.020, *P* = 0.160, partial *η*^2^ = 0.028 |  |
| Change | -0.53 (-1.93, 0.88) | | |  | 0.30 (-1.51, 2.11) | | |  | -0.68 (-2.44, 1.08) | | |  | -2.65 (-4.87, -0.43) | | |  | Interaction: | *F* = 2.018, *P* = 0.160, partial *η*^2^ = 0.028 |  |
| POMS2-FI (Fatigue-Inertia), points |  |  |  |  |  |  |  |  |  |  |  |  |  |  |  |  |  |  |  |
| Baseline | 43.7 | ± | 1.0 |  | 43.1 | ± | 1.1 |  | 43.5 | ± | 1.8 |  | 44.2 | ± | 1.1 |  | LTP: | *F* = 0.023, *P* = 0.880, partial *η*^2^ = 0.0003 |  |
| Post-intervention | 42.5 | ± | 1.0 |  | 42.9 | ± | 1.5 |  | 42.8 | ± | 1.9 |  | 42.1 | ± | 1.2 |  | Physical activity intervention: | *F* = 0.589, *P* = 0.445, partial *η*^2^ = 0.008 |  |
| Change | -1.21 (-3.06, 0.64) | | |  | -0.15 (-2.28, 1.98) | | |  | -0.74 (-2.28, 0.81) | | |  | -2.06 (-3.80, -0.32) | | |  | Interaction: | *F* = 1.646, *P* = 0.204, partial *η*^2^ = 0.023 |  |
| POMS2-TA (Tension-Anxiety), points |  |  |  |  |  |  |  |  |  |  |  |  |  |  |  |  |  |  |  |
| Baseline | 43.5 | ± | 1.2 |  | 45.2 | ± | 1.2 |  | 47.2 | ± | 2.4 |  | 47.2 | ± | 1.8 |  | LTP: | *F* = 0.063, *P* = 0.803, partial *η*^2^ = 0.001 |  |
| Post-intervention | 44.9 | ± | 1.1 |  | 45.3 | ± | 1.3 |  | 44.3 | ± | 1.5 |  | 45.4 | ± | 2.0 |  | Physical activity intervention: | *F* = 2.549, *P* = 0.115, partial *η*^2^ = 0.035 |  |
| Change | 1.42 (-0.64, 3.48) | | |  | 0.15 (-2.67, 2.97) | | |  | -2.89 (-6.43, 0.64) | | |  | -1.82 (-4.77, 1.12) | | |  | Interaction: | *F* = 0.506, *P* = 0.479, partial *η*^2^ = 0.007 |  |
| POMS2-VA (Vigor-Activity), points |  |  |  |  |  |  |  |  |  |  |  |  |  |  |  |  |  |  |  |
| Baseline | 50.8 | ± | 1.6 |  | 51.7 | ± | 1.9 |  | 52.5 | ± | 3.3 |  | 53.4 | ± | 1.8 |  | LTP: | *F* = 0.017, *P* = 0.895, partial *η*^2^ = 0.0002 |  |
| Post-intervention | 54.1 | ± | 1.8 |  | 53.6 | ± | 2.3 |  | 54.9 | ± | 2.4 |  | 57.1 | ± | 2.7 |  | Physical activity intervention: | *F* = 0.347, *P* = 0.558, partial *η*^2^ = 0.005 |  |
| Change | 3.32 (0.73, 5.90) | | |  | 1.90 (-2.62, 6.42) | | |  | 2.42 (-2.92, 7.76) | | |  | 3.65 (0.26, 7.04) | | |  | Interaction: | *F* = 0.532, *P* = 0.468, partial *η*^2^ = 0.008 |  |
| POMS2-F (Friendliness), points |  |  |  |  |  |  |  |  |  |  |  |  |  |  |  |  |  |  |  |
| Baseline | 49.6 | ± | 1.8 |  | 52.6 | ± | 2.0 |  | 54.8 | ± | 2.4 |  | 54.7 | ± | 2.0 |  | LTP: | *F* = 1.062, *P* = 0.306, partial *η*^2^ = 0.015 |  |
| Post-intervention | 52.8 | ± | 2.1 |  | 51.9 | ± | 2.2 |  | 56.4 | ± | 2.2 |  | 55.5 | ± | 3.3 |  | Physical activity intervention: | *F* = 0.180, *P* = 0.673, partial *η*^2^ = 0.003 |  |
| Change | 3.21 (-0.48, 6.90) | | |  | -0.70 (-5.40, 4.00) | | |  | 1.53 (-1.97, 5.03) | | |  | 0.82 (-3.43, 5.08) | | |  | Interaction: | *F* = 0.412, *P* = 0.523, partial *η*^2^ = 0.006 |  |
| POMS2-TMD (total mood disturbance) score, points |  |  |  |  |  |  |  |  |  |  |  |  |  |  |  |  |  |  |  |
| Baseline | 43.2 | ± | 1.1 |  | 44.1 | ± | 0.9 |  | 43.9 | ± | 1.6 |  | 45.5 | ± | 1.3 |  | LTP: | *F* = 0.839, *P* = 0.363, partial *η*^2^ = 0.012 |  |
| Post-intervention | 42.9 | ± | 1.1 |  | 43.9 | ± | 1.0 |  | 42.9 | ± | 1.4 |  | 42.5 | ± | 1.1 |  | Physical activity intervention: | *F* = 4.729, *P* = 0.003, partial *η*^2^ = 0.063 |  |
| Change | -0.26 (-1.44, 0.92) | | |  | -0.20 (-1.83, 1.43) | | |  | -1.05 (-2.54, 0.43) | | |  | -2.94 (-4.89, -1.00)^⁑^ | | |  | Interaction: | *F* = 1.672, *P* = 0.200, partial *η*^2^ = 0.023 |  |
| BDI-Ⅱ, points |  |  |  |  |  |  |  |  |  |  |  |  |  |  |  |  |  |  |  |
| Baseline | 7.79 | ± | 1.54 |  | 6.50 | ± | 1.10 |  | 9.42 | ± | 1.54 |  | 8.71 | ± | 1.22 |  | LTP: | *F* = 0.00003, *P* = 0.996, partial *η*^2^ = 0.0000005 |  |
| Post-intervention | 7.16 | ± | 1.75 |  | 7.10 | ± | 1.35 |  | 7.89 | ± | 1.53 |  | 6.18 | ± | 1.00 |  | Physical activity intervention: | *F* = 4.558, *P* = 0.036, partial *η*^2^ = 0.062 |  |
| Change | -0.63 (-1.50, 0.24) | | |  | 0.60 (-0.87, 2.07) | | |  | -1.53 (-3.53, 0.48) | | |  | -2.53 (-4.98, -0.08)^⁑^ | | |  | Interaction: | *F* = 1.737, *P* = 0.192, partial *η*^2^ = 0.024 |  |
| Supplemental Table 3. Effects of LTP ingestion and physical activity intervention on fatigue and mood status after excluding three participants who were taking angiotensin II receptor blockers and rarely participated in the supervised sessions (n = 75). Data are presented as mean ± standard error for baseline and post-intervention and mean (95% confidence interval) for changes at 8 weeks. Placebo, placebo without physical activity intervention; LTP, lactotripeptide without physical activity intervention; placebo + PA, placebo with physical activity intervention; LTP + PA, lactotripeptide with physical activity intervention; POMS2, Profile of Mood States second edition; BDI-II, Beck Depression Inventory second edition. ^⁑^*P* < 0.05 vs. LTP group. | | | | | | | | | | | | | | | | | | | |

| Week | Time | Intensity | Borg scale |
| --- | --- | --- | --- |
| 1 | 30 min | 60% of estimated maximal heart rate | 11 |
| 2 | 35 min | 65% of estimated maximal heart rate | 13 |
| 3 | 35 min | 70% of estimated maximal heart rate | 13 |
| 4 | 40 min | 70% of estimated maximal heart rate | 13 |
| 5, 6 | 40 min | 75% of estimated maximal heart rate | 15 |
| 7, 8 | 45 min | 75% of estimated maximal heart rate | 15 |
| Supplemental Table 4. Exercise intensities and durations during the supervised session. | | | |

| Stage | Details |
| --- | --- |
| 1 | Increase MVPA by 90 min/week and steps by 12,000 steps/week from baseline |
| 2 | Increase MVPA by 105 min/week and steps by 14,000 steps/week from baseline |
| 3 | Increase MVPA by 120 min/week and steps by 16,000 steps/week from baseline |
| 4 | Increase MVPA by 135 min/week and steps by 18,000 steps/week from baseline |
| Supplemental Table 5. Goals of MVPA at home. MVPA, moderate- to vigorous-intensity physical activity. | |
